# Analysis of Phthalate Migration to Food Simulants in Plastic Containers during Microwave Operations

**DOI:** 10.3390/ijerph110100507

**Published:** 2013-12-30

**Authors:** Miriany A. Moreira, Leiliane C. André, Zenilda L. Cardeal

**Affiliations:** 1 Departamento de Química, ICEx, Universidade Federal de Minas Gerais; Av. Antônio Carlos 6627, Pampulha, 31270-901, Belo Horizonte, MG, Brazil; E-Mail: miriany_qui@yahoo.com.br; 2 Departamento de Análises Clínicas e Toxicológicas, FAFAR, Universidade Federal de Minas Gerais, Av. Antônio Carlos, 6627, Pampulha, 31270-901, Belo Horizonte, MG, Brazil; E-Mail: leiliane@farmacia.ufmg.br

**Keywords:** phthalates migration, cold solid phase microextraction, gas chromatography/mass spectrometry, food simulant, dibutylphthalate, benzylbutylphthalate

## Abstract

Phthalates used as plasticizers in the manufacture of household containers can potentially be transferred to foods that are stored or heated in these plastic containers. Phthalates are endocrine disruptor compounds (EDC) and are found in very low concentrations in foods, thus, highly sensitive analytical techniques are required for their quantification. This study describes the application of a new method developed for analyzing the migration of dibutylphthalate (DBP) and benzylbutylphthalate (BBP) from plastic food containers into liquid food simulants. This new method employs the technique of solid phase microextraction cooled with liquid nitrogen. The analysis was conducted by gas chromatography/mass spectrometry (GC/MS) using a polyacrylate fiber. Ultrapure water was used as a simulant for liquids foods, and both new and used plastic containers were placed in a domestic microwave oven for different periods of time at different power levels. The limits of detection for DBP and BBP were 0.08 µg/L and 0.31 µg/L, respectively. BBP was not found in the samples that were analyzed. DBP was found in concentrations ranging from <LOQ to 7.5 µg/L. In general, an increase in migration was observed in containers that were used for a prolonged time, which correlated with increasing heating time.

## 1. Introduction

Phthalate diesters are widely used as plasticizers to increase the flexibility of plastics that are used in the manufacturing of kitchen utensils and food containers [1]. These phthalates are not chemically bonded to the polymeric matrix, which may allow the migration of these chemicals into food substances [2]. Another source of food contamination is through contact with packaging printing inks that contain phthalates [3]. These compounds are classified as endocrine disruptors (EDC) and can affect the endocrine system, which consequently affects vital functions in living organisms [4]. Dibutylphthalate (DBP), bis-(2-ethylhexyl) phthalate (DEHP), benzylbutylphthalate (BBP), and di-*n*-octylphthalate (DOP), among others, are examples of phthalates [5]. The greatest concern regarding EDC is chronic exposure. Phthalates such as DBP have been associated with toxicity to the neural, reproductive and developmental systems [6]. Studies in animals have shown that phthalates can cause alterations to the kidneys and the liver, fetal malformation and fertility impairments [6,7,8]. In humans, exposure to phthalates such as DEHP and DBP can alter human sperm motility [9]. Other studies have also shown a correlation between endocrine disruptors and diseases related to the endocrine system. A study by Kim *et al.* demonstrated a correlation between the amount of DEHP in plasma samples and advanced stage endometriosis in women [10]. A study by Meeker and Ferguson suggested associations between phthalates and altered thyroid hormone levels [11]. The European Food Safety Authority (EFSA) has established the tolerable daily intake (TDI) for phthalates at 0.01 mg/kg body weight per day for DBP, 0.5 mg/kg body weight per day for BBP, and 0.05 mg/kg body weight per day for DEHP based on toxicological studies [12,13,14]. Phthalates are contaminants found in many foods, such as olive oil, wine, and milk [15,16,17]. These compounds are present at low concentrations in foods, which necessitates the development of highly sensitive analytical techniques for their quantification. Sample pretreatment is necessary to extract and concentrate phthalates and to improve analytical sensitivities. Techniques such as solid phase extraction (SPE), single drop microextraction (SDME), dispersive liquid–liquid microextraction (DLLME), air-assisted liquid–liquid microextraction (AALLME), liquid phase microextraction (LPME), and solid phase microextraction (SPME) may be used for sample pretreatment. SPME has many advantages and is applied mainly when gas chromatography is used. SPME consists of the use of a fiber for the extraction and concentration of an analyte present in a sample. Various types of fiber materials can be used, such as polydimethylsiloxane and polyacrylate. The extraction process consists of the adsorption/absorption of the analyte in a fiber. This process is exothermic, and cooling the fiber accelerates the transfer of the analyte to the fiber. The cooling of the fiber was initially evaluated by Ghiasvand *et al.*, who used carbon dioxide to cool the fiber [18]. A new cooling system was developed by Menezes *et al.* using liquid nitrogen to cool the fiber [19]. In these cooling systems, there was an increase in the extraction efficiency of the analytes. The sample can be heated simultaneously with fiber cooling to allow the release of the analyte. Although they are antagonistic processes, the heating of the sample does not nullify the cooling of the fiber. The fiber is subsequently inserted into the GC injector for the desorption of analytes. Several studies have attempted to determine the migration magnitude of chemical substances present in food packaging materials. A study conducted by Gonzales-Castro *et al.* found concentrations of DBP and DOP that migrated from plastic containers into food simulants of 0.023 µg/L and 0.664 µg/L, respectively [20]. Kueseng *et al.* determined that the concentration of DEHP migrating from curry packaging into curry was 0.61 µg/L [21]. Several studies have been conducted on the migration of phthalates, but little is known about the migration of these compounds from food packaging when they are subjected to different heating conditions in a microwave oven. Thus, this study conducted a quantitative analysis of the migration of dibutylphthalate and benzylbutylphthalate into a food simulant placed in plastic containers when these were subjected to different heating conditions. Water was used to simulate liquid foods with a pH value of more than five.

## 2. Materials and Methods

### 2.1. Materials

The DBP and BBP standards that were used in this study were obtained from Sigma-Aldrich (St. Louis, MO, USA). A stock solution at the concentration of 2.00 g/L per compound was prepared in HPLC grade ethanol purchased from J.T. Baker (Xalostoc, Edo. de Mex., Mexico). The polyacrylate fiber that was used in the experiment was obtained from Supelco (Bellefonte, PA, USA), and ultrapure water was generated using an Elga Purifier, model Classic Purelab UVMK2 (High Wycombe, UK).

### 2.2. Extraction Method

**Figure 1:**
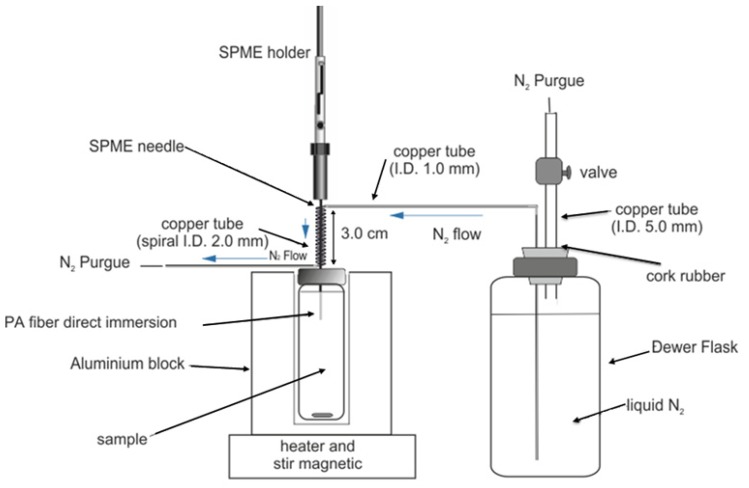
Schematic of the CF-SPME device.

The analytes were extracted by direct immersion extraction using an 85 μm polyacrylate fiber [22]. The extraction was conducted using a cold fiber solid phase microextraction (CF-SPME) [19]. The cryogenic system used in this study was cooling on the outside of the fiber, as shown in Figure 1. A copper tube with a length of 70 cm, O.D. of 2.4 mm, and I.D. of 1.6 mm was used to transfer liquid nitrogen from a Dewar flask to the SPME device. One end of the tube was inserted into the Dewar flask through a rubber stopper, and the other end, composed of a 3 cm- spiral with a 2 mm I.D., held the needle of a manual SPME holder containing an 85 μm polyacrylate (PA) fiber. The rubber stopper (4 mm top diameter and 3 mm bottom diameter) was used to cap the Dewar flask containing 0.5 L of liquid nitrogen. Another copper tube (10 cm length, 6.4 mm O.D. and 4.7 mm I.D.) was used as a valve to control the nitrogen pressure in the Dewar flask; 0.5 L of liquid nitrogen was sufficient for 3 h of cooling. By closing the valve, the liquid nitrogen evaporated slowly and passed through the spiral at a constant rate, absorbing heat from the manual SPME holder and, hence, the fiber. When the valve was open, the nitrogen was purged from the Dewar flask and no longer passed through the spiral, thus terminating the cooling process. For the extraction, the cooled fiber was immersed in 22 mL Pyrex vials sealed with silicone/PTFE septa and aluminum caps containing the sample. The vial was placed in an aluminum block with a controlled temperature and constant stirring.

The fiber was pre-conditioned for 1 h at 280 °C according to the manufacturer’s instructions. For compound extraction, the sample (20.0 mL) was placed in a 22 mL vial and subjected to continuous stirring with a magnetic stirring bar placed within the vial. A multivariate optimization of the extraction methodology was used to reduce the number of assays. The study of variables considered significant for the experiment was conducted using a factorial design of 2^3^. For this experiment, the extraction time, extraction temperature and added salt concentration were varied at two levels selected for the factorial design: 10 and 30 min, 25 °C and 65 °C, and 0% and 10% (w/v) of NaCl. The central point was analyzed in triplicate with the following values for the variables: extraction time, 20 min; extraction temperature, 45 °C; and salt concentration, 5%. The standard solutions that were used in the design of the experiments were at the concentration of 100 µg/L per compound. The fiber was subsequently placed in the gas chromatograph for compound desorption at 250 °C for 2 min. 

### 2.3. Chromatographic System

The analysis of phthalates in the water that was used as a simulant liquid food was conducted using a gas chromatographic system coupled to a mass spectrometer equipped with an ion trap analyzer (Finnigan Trace GC/PolarisQ, Thermo, Austin, TX, USA). Chromatographic analysis was conducted via injection in splitless mode for 2 min using a HP-5MS Agilent column (30 m × 0.25 mm × 0.25 µm) with an injector temperature of 250 °C and a helium flow rate of 1.2 mL/min. The temperature ramp began at 105 °C for 1 min, increased to 180 °C at a rate of 3 °C/min, maintained for 4 min, further increased to 290 °C at a rate of 10 °C/min, and then maintained for 0.5 min. The total running time was 41.5 min. The analysis was conducted in selected ion monitoring (SIM) mode using electron ionization (EI) with an energy of 70 eV. The mass/charge (*m/z*) ratios that were monitored were 149, 150 and 41 for DBP and 206, 149 and 91 for BBP.

### 2.4. Analysis Conditions

To evaluate the migration of phthalates into liquid food, ultrapure water was used as a simulant according to Brazilian legislation. The European legislation EU Regulation 10/2011 [23] considers ethanol 10% (v/v) to be a simulant for liquid food; however, the Brazilian Health Ministry document No. 26/MS/SVS was followed and it specifies that water is an adequate simulant for liquid foods possessing pH values more than 5 [24]. 

This study tested ten polypropylene (PP) containers of different sizes that were suitable for use in microwave ovens. All of the containers studied were from different brands. Five of these containers were newly purchased for this study, and the other five were containers that had been exposed to long-term use of approximately one year. Plastic containers used in the microwave ovens tend to undergo small deformations with time due to the repeated heating of the material, hence, the used containers already contained some small deformations. The containers were filled with ultrapure water to 90% of their total volumes. Subsequently, they were placed in a domestic microwave oven (700 W power) where the water was then heated at various powers (high, 700 W; medium, 350 W; and low, 210 W) and heating durations. The migration of phthalates into the simulant was evaluated at these operating powers at heating durations of 1, 3, 5, and 7 min. For low power, the migration was evaluated at heating durations of 10, 15, 20, and 30 min. The power and heating duration conditions were chosen based on the conditions that are commonly used in everyday life, where the high power level is used for cooking food, the medium power level is used for heating food, and the low power level is used for thawing food, which requires a longer heating period. Samples in which the phthalate concentrations were beyond the range of the calibration curve were diluted to 50% with ultrapure water. The characteristics of the containers that were used are shown in Table 1.

**Table 1:** Characteristics of the plastic containers that were used.

Plastic Containers	Volume (mL)	Characteristics
A ^1^	750	colorless, size: 10.5 cm (dm) × 11.0 cm (h)
B ^1^	500	red translucent, size: 10.5 cm (dm) × 6.2 cm (h)
C ^1^	600	colorless, size: 13.2 cm × 13.2 cm × 4.8 cm
D ^1^	1,200	colorless, size: 12.6 cm × 12.6 cm × 11.0 cm
E ^1^	480	colorless, size: 12.5 cm × 9.3 cm × 5.3 cm
F ^2^	600	colorless, size: 13.0 cm × 13.0 cm × 4.7 cm
G ^2^	1,000	colorless, size: 13.7 cm × 10.0 cm × 2.7 cm
H ^2^	600	colorless, size: 11.2 cm (dm) × 6.6 cm (h)
I ^2^	1,200	colorless, size: 22.0 cm × 15.0 cm × 4.6 cm
J ^2^	300	colorless, size: 13.8 cm (dm) × 7.9 cm (h)

Notes:^ 1^ new containers; ^2^ containers that were used for prolonged periods of time.

Phthalates are contaminants that are present in various matrices such as water and air. Additionally, it is possible that the plastic containers are cross contaminated with DBP from the laboratory through contact with plastic materials. To avoid contamination, all glassware used was washed in a special manner using Extran^®^ (Merck, Darmstadt, Germany) followed by ethanol. A sample blank was prepared and analyzed periodically to determine if there was background contamination. A low concentration of DBP was detected in the sample blank. This concentration was smaller than the limit of quantification (LOQ); nevertheless, it was considered when determining the compound in the samples and during the validation process.

## 3. Results and Discussion

### 3.1. Extraction Method

**Figure 2:**
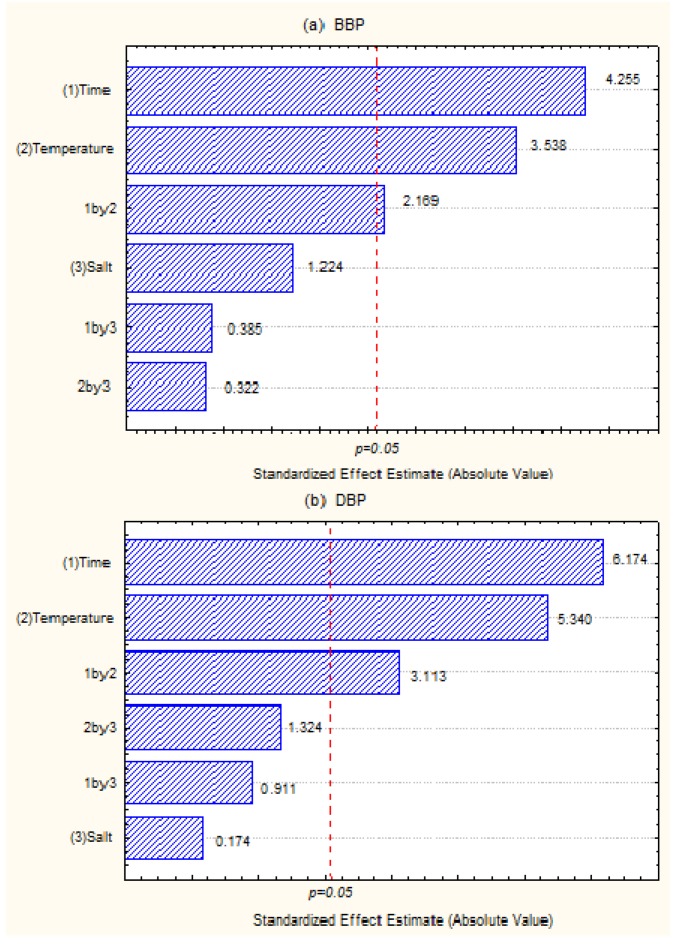
Pareto charts for (**a**) BBP and (**b**) DBP obtained from the 2^3^ factorial experiment design.

Pareto charts in a 2^3^ factorial design were used to evaluate the significant parameters in the extraction process. The optimization standard solution concentrations were 100 µg/L. Thus, all conditions tested were able to produce peaks in the chromatographic analysis with an area that would allow high detection. Graphs were obtained using the Statistica v.8 software (Hewlett-Packard, Palo Alto, CA, USA). From the data obtained in the graphs (as shown in Figure 2), it was observed that time, temperature, and the interactions between these two variables had significant effects on the extraction step of the two phthalates at a 95 % confidence interval. The graphs demonstrated that the time and temperature variables followed the same trend for the two compounds. Therefore, larger areas were obtained using an extraction duration of 30 minutes and an extraction temperature of 65 °C due to larger chromatographic signals. Thus, these conditions were used in the analytical procedure with no added salt.

### 3.2. Analytical Validations

The parameters of merit were evaluated according to EURACHEM guidelines [25]. The limit of detection (LOD), LOQ, intra- and inter-assay precisions and linearity were determined. The linearity ranges that were observed were 0.2 to 6.0 µg/L for DBP and 0.5 to 6.0 µg/L for BBP. The calibration curve was constructed from the analysis of standards at six different concentrations (0.2, 1.0, 2.0, 4.0, 5.0 and 6.0 µg/L for DBP and 0.5, 1.0, 2.0, 4.0, 5.0 and 6.0 µg/L for BBP) in triplicate for each point. To evaluate the linearity of the curve obtained, the following statistical tests were applied: normality of residues (Ryan-Joiner Test), independence of residues (Durbin-Watson Test), homoscedasticity of residues (Brown-Forsythe Test), significance regression, and deviation from linearity based on an analysis of variance (ANOVA) test [26]. The tests performed demonstrated that the residues followed a normal distribution were independent, and possessed homoscedasticity. The ANOVA test indicated that the regression was significant and that there was no deviation from linearity. Therefore, the linear regression was used for the quantification of phthalates. The equations and the determination coefficients that were obtained from linear regression are shown in Table 2. 

**Table 2:** Equations and determination coefficients for DBP and BBP.

Compound	Linear Range (µg/L)	Equation	SD Intercept	SD Slope	Coefficient of Determination (R^2^)
DBP	0.2 to 6.0	y = 55,438x + 14,941	5,007	1,354	0.9905
BBP	0.5 to 6.0	y = 29,500x – 4,882.9	3,322	897	0.9854

The LOQs were determined according to EURACHEM, and were considered the lowest points on the calibration curves. LOD areas were obtained from the calibration curves according to the mean blank response plus three times the standard deviation. Accuracy tests were evaluated at two different concentrations along the curve, which were 1.0 and 5.0 µg/L. Table 3 displays the LODs, the LOQs, and the accuracies that were obtained using this method. When compared with other studies of food simulants, the detection limits that were found in this study were similar. Using SDME and GC-FID, Batlle and Nerín, obtained a DBP detection limit of 0.03 µg/L when distilled water was used as the food simulant and a detection limit of 0.16 µg/L when a 3% w/v acetic acid solution in distilled water was used as the food simulant [27]. Jen and Liu, using hollow fiber microdialysis enrichment system and HPLC/UV, obtained a LOD of 0.4 µg/L in aqueous solutions for DBP when they evaluated the migration of phthalates from plastic containers under heating conditions [28]. 

The ANOVA test indicated that the intra- and inter-assay precisions were statistically equal to a concentration of 5.0 µg/L for both DBP and BBP. At a concentration of 1.0 µg/L, the ANOVA test indicated that the means and standard deviations of the intra- and inter-assay precisions were different for the two phthalates, with the analytical curve showing a greater variability at lower concentrations. The coefficients of variation that were observed in this study were relatively large compared to the deviations that are reported in the literature, which is also dependent on the concentration range that is studied, where higher concentrations exhibit lower variations.

**Table 3:** Precision, limits of detection, and quantification of DBP and BBP (n = 10).

Compound	RSD—Intra-assay		RSD—Inter-assay		LOD µg/L	LOQ µg/L
	1.0 µg/L	5.0 µg/L	1.0 µg/L	5.0 µg/L		
	%		%			
DBP	11.7	7.6	11.9	8.2	0.08	0.2
BBP	16.2	11.0	22.3	13.9	0.31	0.5

A study by Cavaliere *et al.* reported deviations of 9.5% and 3.0% for DBP and BBP, respectively, in samples of olive oil at concentrations of 0.5 mg/kg [15]. Rios *et al.* determined accuracies of 4.7% and 6.1% for DBP and BBP, respectively, in samples of olive oil at concentration of 1.0 mg/kg [29]. A coefficient of variation of up to 20% is considered suitable for food analysis; hence, the deviation that is obtained should be acceptable [25,30].

### 3.3. Concentration of Phthalates due to Migration from Plastic Containers Heated in a Microwave Oven

The plastic containers that were used for the migration analysis were divided into two groups: five new containers (Group I) and five containers that had already been used for a prolonged period of time (Group II) of approximately one year. The presence of BBP was not observed in any of the containers that were evaluated. The results that were obtained for DBP are presented below.

#### 3.3.1. GROUP I: New Containers

**Figure 3:**
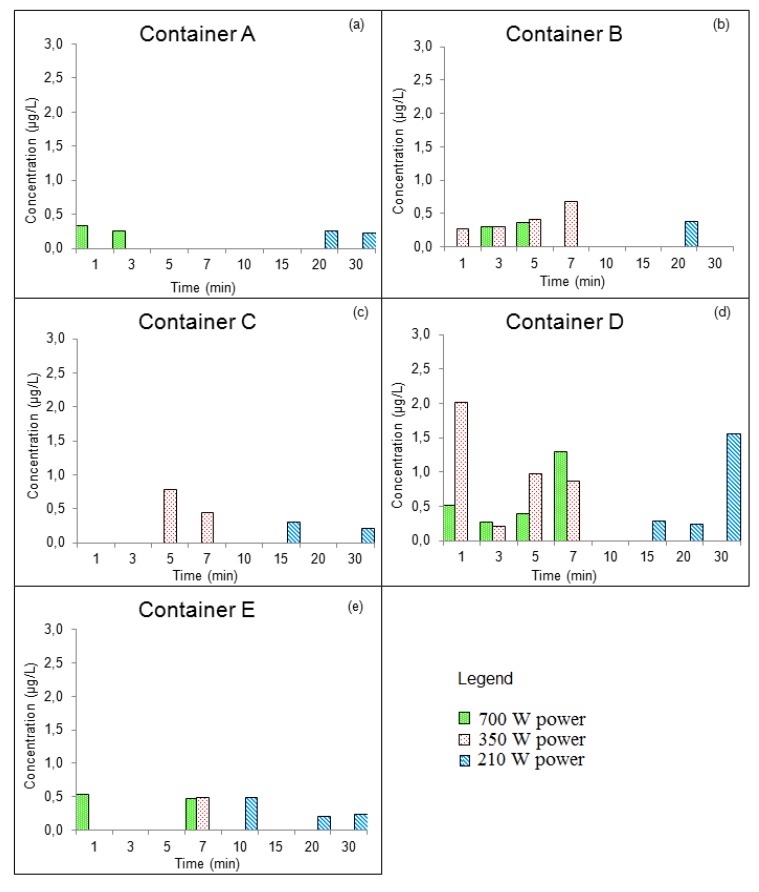
Concentrations of DBP that migrated into Group I food simulant (**a**) container A, (**b**) Container B, (**c**) Container C, (**d**) Container D, and (**e**) Container E.

The concentrations of DBP that migrated into the food simulant at different conditions are displayed in Figure 3a‒e.

#### 3.3.2. GROUP II: Containers that Were Used for Prolonged Periods of Time

**Figure 4:**
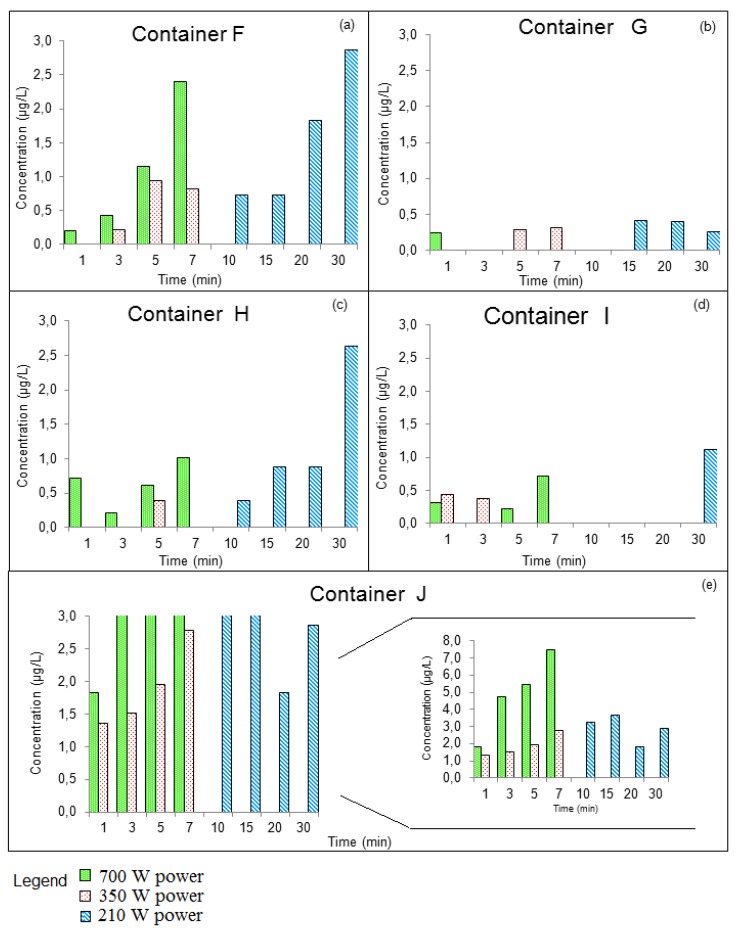
Migrated DBP concentrations in the Group II food simulant containers (**a**) container F, (**b**) Container G, (**c**) Container H, (**d**) Container I, and (**e**) Container J.

The concentrations of DBP that migrated into the food simulant under different exposure conditions are shown in Figure 4a‒e.

The results observed for the DBP concentrations in the Group I containers ranged from <LOQ to 2.0 µg/L, while the DBP concentrations in Group II containers ranged from <LOQ to 7.5 µg/L. The total of all measurements of DBP concentrations that were obtained in the two groups were compared by ANOVA. The results showed that there was a difference in the average concentration of the two groups, with the average DBP concentration found in Group II containers being larger than that found in Group I containers. Therefore, it can be concluded that the release of phthalates from plastic containers increases with continued use of the containers.

**Figure 5:**
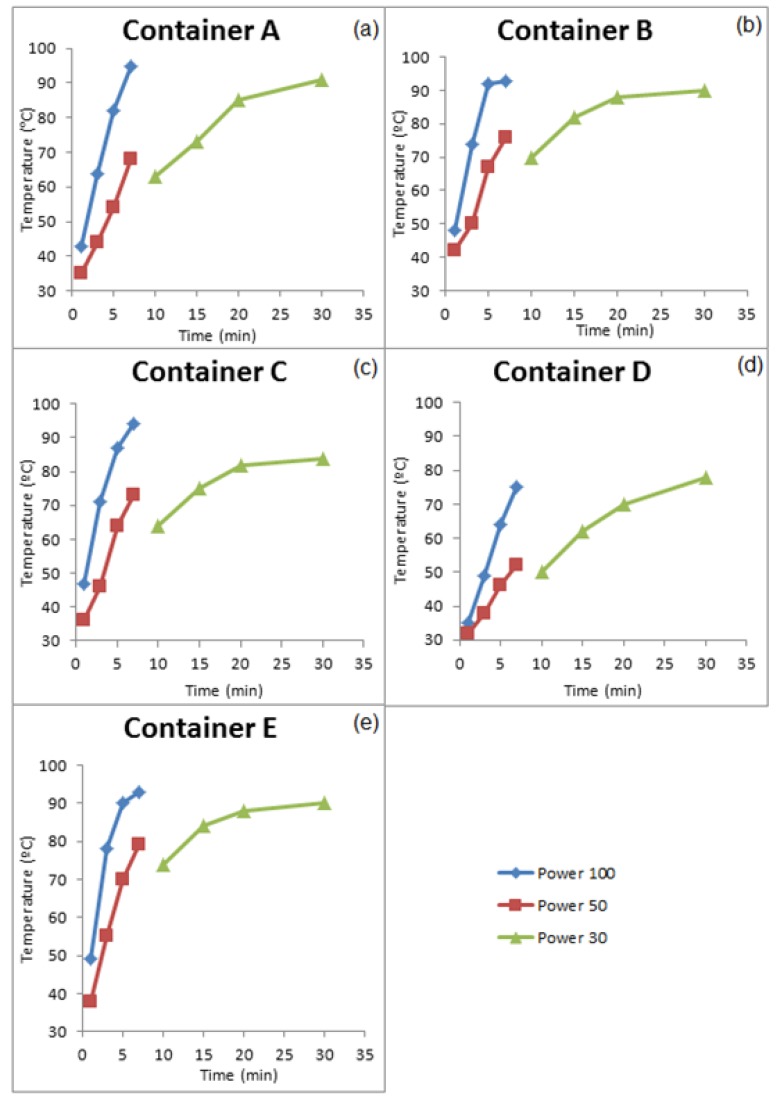
Temperatures of simulants measured for times and powers studied for Group I containers (**a**) Container A, (**b**) Container B, (**c**) Container C, (**d**) Container D, and (**e**) Container E.

**Figure 6:**
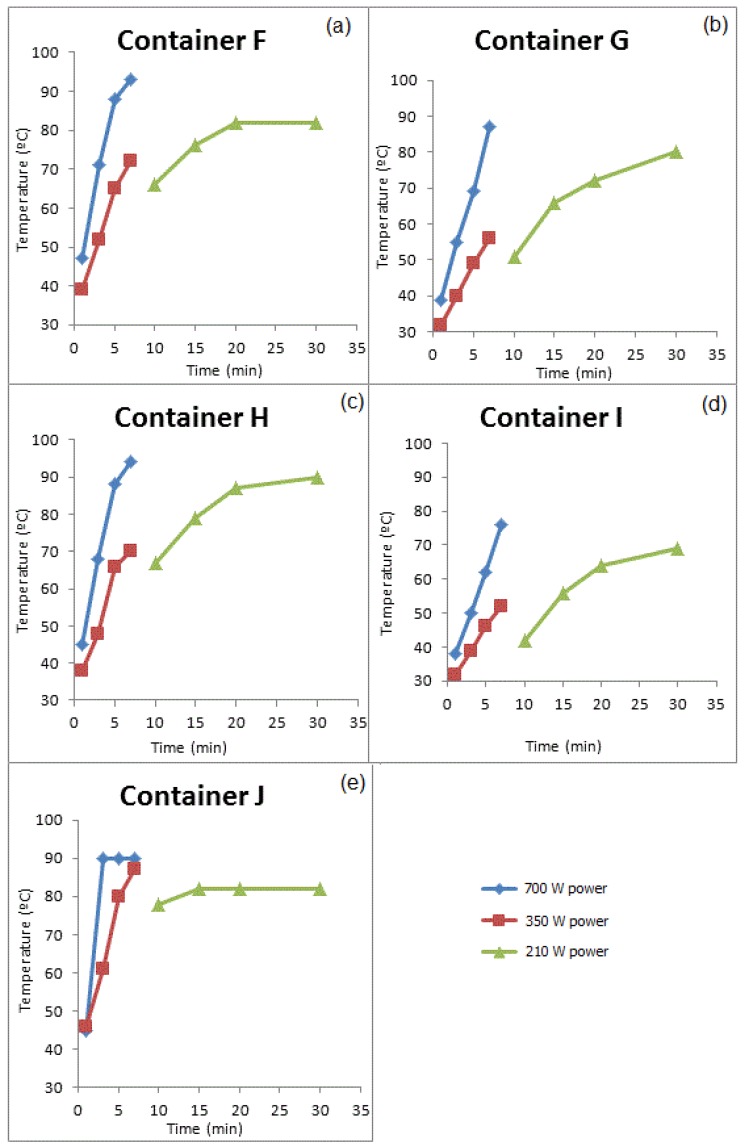
Temperatures of simulants measured for times and powers studied for Group II containers (**a**) container F, (**b**) Container G, (**c**) Container H, (**d**) Container I, and (**e**) Container J.

All containers were exposed to the same heating conditions; however, it is known that microwave irradiation often leads to non-homogeneous heating, which also depends on the shape and size of the containers. To evaluate the heating the simulant temperature was measured immediately after heating. A thermometer was placed into the sample, which was shaken to allow for measurement of the mean temperature. The temperatures obtained for the Group I Group II containers are presented in Figure 5 and Figure 6, respectively.

An examination of Figure 3 and Figure 4 shows that, in new containers the increase in heating time influences the increase in temperature; however, there is not a correlation between these factors and the migration of phthalates. Therefore, the DBP concentrations in the Group I containers were independent of the heating duration or the power conditions that were used for the containers that were evaluated. The variation in the concentration of DBP in the simulant was random. The highest concentration of DBP (2.0 µg/kg) was observed in container D. However, this values is less than the specific migration limit (SML) for DBP, which is 0.3 mg/kg.

In Group II, different amounts of DBP were observed in the samples. Through the comparison between the migration of phthalate (Figure 4) and the temperature obtained in each migration assay (Figure 6), it was observed that the migration of phthalate in the containers that had already been used for a prolonged period of time depended on the temperature achieved during heating and the exposure time of the container to this temperature. The temperature reached depended on the time of exposure to the microwaves, volume and shape of the container and power used. In general, it was observed that, using 750 W and 350 W powers, the temperature increased abruptly with increasing of time. When heating at the 210 W power level, the temperature began high and increased slightly with heating time.

Container J had the smallest volume, 300 mL, and consequently a large temperature variation was observed in the interval from 1 to 3 min at the 700 W power level. Heating at this power for 3 min resulted in a final temperature of 90 °C. This temperature remained constant for the longest time; thus the simulant remained in contact with the container at a high temperature for a longer time which resulted in an increased migration of phthalates to the simulant. At the 350 W power level the temperature variation occurred more gradually and there was an increase in the migration of DBP with the increase in temperature. However, the variation in the concentration of DBP in the sample was lower. At 210 W power, a small variation of temperature was observed for different exposure times, and thus there was not a linear relationship between exposure time and phthalates migrations. 

Containers G and I had large volumes of 1,000 mL and 1,200 mL, respectively. Lower temperatures were obtained when compared to other containers heated at the same power. Consequently, a small variation in the concentration of DBP was observed. Containers F and H have the same volume, 600 mL, but different dimensions. In general, it was observed that an increase in temperature caused an increase in the migration of phthalates into the simulant.

The obtained results suggest that the increase in temperature is the main cause of phthalate migration. Theoretically, containers used in microwaves are inert; however, studies have shown that the migration of various chemical substances may occur during microwave heating. A study by Nerin *et al.* demonstrated that the release of compounds from plastic containers is temperature-dependent. According Nerin, the temperature increase leads to an increase in the decomposition of additives and breakdown of polymer chains which causes the release of chemicals from the hot plastic surface [31]. Other studies indicate that phthalates may be released during microwave heating. Phthalates are not commonly used as plasticizers in PP. However a study by Jen and Liu determined an average concentration DBP of 32 µg/L in PP soup bowls when they were heated for 10 minutes in a microwave oven at a power of 500 W [28]. A study by Gonzales-Castro *et al.* determined a DBP concentration of 0.001 µg/L in aqueous solutions that were heated in the microwave oven [20]. In these two studies, the analysis conditions, such as the heating duration and temperature, were not varied. The results of other studies in which where phthalates were monitored in different types of food are shown in Table 4.

When evaluating the concentration of phthalates, it is essential to consider the characteristics of the food. Phthalates are lipophilic and are found in higher concentrations in fatty foods, such as olive and other oils [15,32]. In this work, DBP concentrations that were similar to the concentrations reported in the analysis of hydrophilic food samples were obtained [17,20].

The plastic containers that are used in microwave ovens are usually made of polypropylene, where the use of plasticizers is not essential and its addition should not be intentional. Therefore, phthalate migration tests are often not required for PP packaging. However, Shen reported the presence of phthalates at concentrations of 1.44 mg/kg for DBP, 0.10 mg/kg for BBP, and 8.72 mg/kg for DEHP in plastic containers that were being used in microwave ovens [33]. These results demonstrate that PP containers can contain plasticizers. A possible source of DBP contamination is the use of the TiCl_4_/dibutylphthalate/Mg(OEt)_2_ catalyst in the manufacturing of polypropylene. During the polymer manufacturing process, the catalyst may undergo degradation and release this phthalate into the polymer material [34]. Another possible source of contamination could arise from the contact Group II containers with food contaminated with phthalates. Frequent contact with contaminated food could cause contamination of containers over prolonged use. Several studies have shown that many foods are contaminated with phthalates (Table 4). This hypothesis has been raised, because higher concentrations of phthalates have been found in containers with long-term use when compared to new containers. Although the concentrations of DBP in Group II containers were larger than Group I containers, it was observed that the concentration of DBP was much smaller than 0.3 mg/kg in all containers, which is the legal specific migration limit. The legislation control for regulating phthalates in various routes of exposure is not established for all compounds in this class because more studies are needed for many phthalates. In drinking water, the US EPA stipulates a maximum contamination level (MCL) of 6.0 µg/L for DEHP [35]. However, the MCL has not been estimated for other phthalates. In this work, the maximum concentration of DBP found in the water samples that were heated in a microwave oven was 7.5 µg/L. If the value set by the US EPA for DEHP was extended to the entire class of phthalates, the value found in this study would exceed the MCL. Legislation for food is less strict for the migration of phthalates used in plastic packaging. The Brazilian Institution ANVISA, which has reference guidelines from the Food and Drug Administration and documents from the European Union, established a specific migration limit of 0.3 mg of DBP per kilogram of simulant that is in contact with food packaging [36]. The SML of BBP is 30.0 mg/kg. Hence, following the Brazilian legislation from ANVISA, the concentrations that were found in this work would be within the allowed amount. With regards to health effects, many types of foods can be contaminated by phthalates during the manufacturing process, resulting in even higher concentrations of phthalates in foods. Thus, there is a great need for further studies to evaluate food contamination by phthalates.

**Table 4:** Phthalate concentrations that have been reported in the literature.

Compounds	Concentration (µg/kg)	Samples	Method of Extraction	Instrumental	LOD	LOQ	Reference
DBP	7.30 to 50.3	Commercial whole milk	SPE (C18)	GC/MS	0.09		[37]
BBP	1.11 to 2.93				0.12	—	
DEHP	15.1 to 27.2				0.06		
DBP	<LOD to 490	Olive oil	Gel permeation chromatography	GC-MS/MS			[15]
BBP	<LOD to 1,750						
DEHP	<LOD to 4,700				—	—	
DEHP	22.8 to 270.3	Cereals and legumes, Meat based, fish based, dairy, vegetables, condiments, fresh fruit, bread	LLE	GC-FID	—	—	[38]
DBP	10.2 to 142.8						
DBP	<LOD to 244 (µg/L)	Wines	SPE	GC/MS	18 (µg/L)	29 (µg/L)	[16]
BBP	<LOD to 269 (µg/L)				18 (µg/L)	29 (µg/L)	
DEHP	<LOD to 276 (µg/L)				15 (µg/L)	24 (µg/L)	
DBP	4.07 to 9.79	Cow milk	HS-SPME	GC/MS	0.02 a 072		[17]
BBP	<LOD				0.23 a 4.7	—	
DEHP	8.40 to 282.90				0.31 a 3.3		
DBP	0.001 to 0.003	Water (food simulant)	LLE	GC/MS, HPLC/UV-VIS and HPLC fluorescence	2.0 ×10^−3^	5.0 ×10^−3^	[20]
DBP	32 and 24 (µg/L)	Water released from disposable PP soup bowl and PVDC wrap film	Hollow fiber microdialysis enrichment system	HPLC/UV	0.4 (µg/L)	—	[28]
DEHP	77 to 1643	Commercial vegetable Oils	Direct injection of thediluted oil	GC/MS	10	40	[32]
DBP	22 to 360				10	40	
DBP	<LOD to 175	Olive oil	SPME	GC/MS/MS	30	—	[29]
BBP	87 to 211				30		
DEHP	198 a 840				30		
DBP	<LOD to 9,840	Plastic containers	Sonication-assisted extraction	GC/MS	10	—	[33]
BBP	<LOD to 9,980						
DEHP	<LOD to 1.76 × 10^6^						
DBP	<LOD	Bottled mineral water	AALLME	GC-FID	0.37 (µg/L)	—	[39]
DEHP	0.26 to 0.32 (µg/L)				0.75 (µg/L)		
DBP	0.32 to 0.51 (µg/L)	Bottled mineral water	LPME	GC/MS	0.005 (µg/L)	—	[40]
BBP	<LOD						
DEHP	0.57 to 0.65 (µg/L)				0.01 (µg/L)		
					0.02 (µg/L)		
DBP	<LOD	Bottled mineral water	DLLME	GC/MS	0.005 (µg/L)	—	[41]
BBP	<LOD				0.002 (µg/L)		
DEHP	<LOD				0.005 (µg/L)		
DBP	0.2 to 7.5	Water (food simulant)	SPME	GC/MS	0.08 (µg/L)	0.2 (µg/L)	This study
BBP	<LOD				0.31 (µg/L)	0.5 (µg/L)	

## 4. Conclusions

The method that was developed in this study is suitable for the quantification of phthalates present at low concentrations in liquid samples. The main advantage of using SPME in comparison with other extraction techniques is that organic solvents are not required for sample preparation. EDC are toxic substances even at low concentrations. Therefore, lower quantification limits are required for the evaluation of phthalates in samples that contain small amounts of them and other EDC. The use of cold SPME allowed for the quantification of phthalates that were found in low concentrations in the samples because of increased efficiency in the extraction step. Cold SPME can be used for various compounds and is a technique that can be potentially applicable for the study of EDC and other organic compounds that are present in trace concentrations in different matrices. Phthalates were found in all PP containers. The analyses performed demonstrated that there is a greater migration of phthalates in containers with a prolonged time of use. After prolonged use containers possess small deformations and are less resistant to heat, which may allow phthalates to be more easily released. The values of DBP obtained in this study are in accordance with current legislation for the migration of this compound; however, additional studies are required to evaluate the toxic effects of human exposure to these compounds because the microwave oven is just one of several exposure sources. This study also demonstrates that the developed method could be used for real food samples if they possess the characteristics of the simulant that was used.
